# Chemical Profiling, Antiproliferative and Antimigratory Capacity of *Haberlea rhodopensis* Extracts in an In Vitro Platform of Various Human Cancer Cell Lines

**DOI:** 10.3390/antiox11122305

**Published:** 2022-11-22

**Authors:** Katerina Spyridopoulou, Sotiris Kyriakou, Angeliki Nomikou, Angelos Roupas, Antreas Ermogenous, Katerina Karamanoli, Daniela Moyankova, Dimitar Djilianov, Alex Galanis, Mihalis I. Panayiotidis, Aglaia Pappa

**Affiliations:** 1 Department of Molecular Biology & Genetics, Faculty of Health Sciences, Democritus University of Thrace, 68100 Alexandroupolis, Greece; 2 Department of Cancer Genetics, Therapeutics & Ultrastructural Pathology, The Cyprus Institute of Neurology & Genetics, Ayios Dometios, Nicosia 2371, Cyprus; 3 Laboratory of Agricultural Chemistry, School of Agriculture, Faculty of Agriculture, Forestry and Natural Environment, Aristotle University Thessaloniki, 54124 Thessaloniki, Greece; 4 Department of Functional Genetics, Abiotic and Biotic Stress, Agrobioinstitute, Agricultural Academy, 8 Dragan Tzankov Blvd., 1164 Sofia, Bulgaria

**Keywords:** *Haberlea rhodopensis*, resurrection plant, aqueous extract, ethanol extract, antioxidant activity, antimigratory activity, antiproliferative activity, flavonoids, phenolic acids, monoterpenoids, tannins, pigments, soluble proteins, soluble sugars

## Abstract

*Haberlea rhodopensis* is a Balkan endemic plant that belongs to the *Gesneriaceae* family, and is believed to have medicinal use and health-promoting properties. This study aimed to (**i**) prepare aqueous (HAE) and ethanolic (HEE) extracts from the leaves of *H. rhodopensis* from in vitro propagated plants, (**ii**) screen for their potential antiproliferative and antimigratory activities, and (**iii**) chemically characterize both HAE and HEE by identifying compounds which may contribute to their observed bioactivity thereby further supporting their potential use in biomedical applications. The antiproliferative activity of both extracts was assessed against six human cancer cell lines by employing the sulforhodamine-B (SRB) assay. HEE was found to be more potent in inhibiting cancer cell growth as compared to HAE. Therefore, HEE’s antimigratory effects were further studied in hepatocellular carcinoma (HepG2) and non-small cell lung adenocarcinoma (A459) cell lines as they were among the most sensitive ones to its antiproliferative activity. HEE was found to exert significant antimigratory concentration-dependent effects in both cell lines assessed with the wound healing assay. Chemical characterization by UPLC-MS/MS analysis identified that HEE contains higher levels of flavonoids, phenolic compounds, pigments (chlorophyll–/-b, lycopene, and β-carotene), monoterpenoids, and condensed tannins compared to HAE, while HAE, contains higher levels of soluble protein and sugars. Furthermore, HEE demonstrated remarkable antioxidant activity evaluated by the 2,2-diphenyl-1-picrylhydrazyl (DPPH^●^), 2,2-azinobis (3-ethyl-benzothiazoline-6-sulfonic acid) (ABTS^●+^) and ferric reducing/antioxidant power (FRAP) assays. We have obtained comprehensive results highlighting the potential of HEE as a source of bioactive compounds with anticancer properties. Future studies should aim at identifying the chemical constituents responsible for the bioactivities observed, and focus on investigating HEE’s effects, in in vivo preclinical cancer models.

## 1. Introduction

*Haberlea rhodopensis* is a plant of the *Gesneriaceae* family with characteristic lilac flowers that grows in rocky areas and is endemic in the Rhodope Mountains of the Thracian regions of Bulgaria and Greece. *H. rhodopensis* belongs to the group of resurrection plants, which can withstand prolonged drought periods tolerating desiccation, while they quickly resume growth within hours upon rehydration [1]. It is a protected species in Greece and Bulgaria, due to its growth in limited places [2].

The plant leaves have been used in Bulgarian folk medicine for wound healing, detoxification [2], and the treatment of animal diseases [3]. Other plants exhibiting similar desiccation-tolerance properties have been used in the traditional medicine of various ethnic groups for the treatment of the common cold, cough, and bronchitis [4], for their analgesic, hepatoprotective, and anti-epileptic effects [5], as well as for the alleviation of symptoms of influenza and mastitis, for backaches, kidney disorders, hemorrhoids, abdominal pains, scurvy, halitosis, and gingivitis [6,7,8].

Resurrection plants can withstand dehydration by adapting their physiological functions at the molecular level through gene expression regulation [6,9]. It is noteworthy that it has been proposed that there is a distinct pattern of changes that occurs in resurrection plants on the transcript, proteome, and metabolome levels during a dehydration/rehydration cycle [9]. Unique metabolites and compounds have been identified in several resurrection plants that appear to promote and mediate desiccation tolerance and protection against the associated stress [6].

These compounds, exerting a diverse range of bioactivities, possess great potential in biomedical applications as a source of novel health-promoting phytochemicals. Leaf extracts of *H. rhodopensis*, as mixtures of various compounds, have been proven to exert radioprotective effects in vitro and in vivo in New Zealand rabbits [7,10,11,12], immunostimulatory activity in vivo in Wistar rats and humans [13,14], anti-inflammatory activity in vitro [11], and antimicrobial effects in vitro, including antibacterial and antivirus activities [15,16]. Nevertheless, *H. rhodopensis* extracts’ antioxidant activity, is the bioactivity that has attracted the most attention. During desiccation-related stress conditions, it is of utmost significance for the plant to be protected from the dehydration/rehydration processes that induce mechanical, structural, metabolic, and chemical stresses thereby promoting cellular damage [17]. Recruitment of effective antioxidants is among the main protective strategies employed. Thus, *H. rhodopensis* extracts are being examined for their antioxidant efficacy in various in vitro conditions [3,9,10,18,19,20].

Despite the interesting bioactivities reported, there is a paucity of literature on the potential anticancer effects of *H. rhodopensis* extracts. Cell growth studies in HL-60, HL-60/DOX, SKW-3 (leukemia), and MDA-MB-231 (breast cancer) cells, did not reveal any effect up to 500 μg/mL of *H. rhodopensis* methanolic extract [3]. In PC3 cells (prostate cancer), *H. rhodopensis* methanolic extract promoted apoptosis, under H_2_O_2_-induced stress conditions, while it exhibited protective effects in the non-cancerous HEK293 cell line (under the same experimental conditions) [20].

There are different extraction techniques based on the solvent used, capable of producing extracts with different chemical compositions and in different analogies. Although most studies on *H. rhodopensis* focus on methanolic extracts [3,20], the use of aqueous and ethanolic extracts (despite containing fewer compounds than methanolic ones) is also well justified as ethanolic and aqueous extracts are less toxic and more suitable for in vitro bioactivity studies. Therefore, this study aimed to prepare aqueous and ethanolic extracts from the leaves of *H. rhodopensis* and investigate their potential antiproliferative effects by utilizing a panel of human cancer cell lines consisting of A549 (non-small cell lung adenocarcinoma), HepG2 (hepatocellular carcinoma), HT29 and Caco-2 (colorectal adenocarcinomas), as well as PC3 and DU145 (prostate adenocarcinomas). In this in vitro platform, we assayed the growth inhibitory effects exerted by the extracts and proceeded with the most potent ones in investigating the potential antimigratory activity. Notably, in our study, we only utilized extracts from biotechnologically obtained plants, propagated in vitro, since *H. rhodopensis* is a protected species. Finally, we studied the chemical composition of both aqueous and ethanolic *H. rhodopensis* extracts and identified major compounds which could further justify the potential exploitation of *H. rhodopensis*-derived compounds for biomedical applications.

## 2. Materials and Methods

### 2.1. Chemicals and Reagents

Chemicals: acetic acid (A6283), trichloroacetic acid (TCA) (T6399), Trizma base (T4661), sulforhodamine-B (SRB) (230162), aluminum trichloride (237051), sodium acetate (S2889), sulfuric acid 95–97% (10009731), phenol (P1037), hydrochloric acid 37% (H1758), and *n*-butanol (B7906) were purchased from Sigma-Aldrich (St. Louis, MO, USA) unless otherwise stated. Solvents: (chloroform, purity ≥ 99.8% (319988), methanol LC-MS, grade ≥ purity 99.9% (34860), water HPLC grade (34877), acetonitrile HPLC grade, purity ≥ 99.9 (34851), acetone ≥ 99.8% (34580), and formic acid LC-MS grade (85178) were purchased from Honeywell (Medisell Nicosia, Cyprus). Analytical standards: ascorbic acid (ST0800102) and Trolox (ST08003) were purchased from Bioquochem (Asturias, Spain). Catechin (43412), mannose (92683), and linalool (62139) were purchased from Sigma Aldrich (Saint Louis, MO, USA). Gallic acid (4993S), chlorogenic acid (4991S), ferulic acid (4753S), ellagic acid (6075), vanillic acid (6113), caffeic acid (6034S), syringic acid (6011), *p*-coumaric acid (4751S), rosmarinic acid (4957S), 4-hydroxybenzoic acid (6099), protocatechuic acid (6050), 2′-hydroxyflavanone (1180), 7-hydroxyflavanone (1212), 4′-methoxyflavanone (1185), 5-methyxyflavanone (1186), apigenin-7-*O*-glucoside (1004S), luteolin-7-*O*-glucoside (1126S), isorhamnetin (120S), quercetin-3-*O*-rhamnoside (1236S), hyperoside (1027S), myricetin-3-*O*-galactoside (1355S), kaempferol-3-*O*-rutinoside (1053), ipriflavone (1328), and naringin (1129S) were purchased from Extrasynthese (Lyon, France). Bovine Serum Albumin (BSA) (23209) was from Thermofisher (Medisell, Nicosia, Cyprus). The bicinchoninic acid (BCA) Protein Assay Kit (23225) was purchased from Thermo Scientific (Waltham, MA, USA). ABTS Assay Kit (KF01002), 2,2-diphenyl-1-picrylhydrazyl (DPPH) Assay Kit (KF01007), and Total Polyphenol Assay Kit (KB03006) were purchased from Bioquochem (Asturias, Spain). Dulbecco’s Modified Eagle’s Medium (DMEM) and phosphate-buffered saline (PBS) were obtained from Gibco (Thermo Fisher Scientific, Waltham, MA, USA); trypsin, fetal bovine serum (FBS) and penicillin/streptomycin were purchased from Biosera (Boussens, France).

### 2.2. Plant Material and Preparation of Plant Extract

Plants of *H. rhodopensis* were propagated routinely in vitro and adapted in pots under controlled conditions, as previously described [21]. Fully developed leaves from well-hydrated pot plants were detached and air-dried to be used for extraction. For the *H. rhodopensis* aqueous extract (HAE), air-dried leaves (50 mg) were ground in 1.5 mL distilled water and then boiled for 10 min. The samples were centrifuged at 10,000× *g* for 10 min. The supernatant was collected and freeze-dried. For the *H. rhodopensis* ethanol extract (HEE), air-dried leaves (50 mg) were ground in 0.5 mL 70% ethanol. Extraction took place for 48 h at room temperature (RT). The samples were centrifuged at 10,000× *g* for 10 min and the supernatants were collected. The remaining pellet was subjected to additional extraction for 24 h at RT. After additional centrifugation, the supernatant was collected. Both supernatants were combined and dried at 40 °C by SpeedVac. Both HAE and HEE were stored at −20 °C and dissolved in dimethyl sulfoxide (DMSO) before use. Serial dilutions of dissolved extracts were prepared in culture medium for incubating cells with the extracts.

### 2.3. Cell Lines

The human cancer cell lines A549 (non-small cell lung adenocarcinoma, ATCC CCL-185), HepG2 (hepatocellular carcinoma, ATCC HB-8065), HT29 (ATCC HTB-38) and Caco-2 (ATCC HTB-37) (colorectal adenocarcinomas), and PC3 (ATCC CRL-1435) and DU145 (ATCC HTB-81) (prostate adenocarcinomas) were obtained from the American Type Culture Collection (Rockville, MD, USA). All cell lines were maintained under sterile conditions at 37 °C in a humidified atmosphere of 5% CO_2_ and routinely passaged with trypsin. PC3 and DU145 cells were cultured in DMEM-F12 medium whereas the other cell lines were cultured in DMEM. All media were supplemented with 10% fetal bovine serum, penicillin (100 U/mL), and streptomycin (100 µg/mL).

### 2.4. Cell Growth Assay

The cell growth rate was investigated by the sulforhodamine-B (SRB) assay as previously described [22]. The SRB method is a colorimetric assay based on the analysis of cellular protein content that is used for cell density determination [23]. *H. rhodopensis* extracts were dissolved in DMSO. Cells were incubated with serial dilutions of the extracts’ preparations (DMSO concentration ≤ 0.1% *v*/*v*) for either 48 or 72 h, before being stained with SRB. Control cells were incubated with DMSO, whereas unstained samples for each treatment group served as background controls. For the quantification of the optical density, a microplate reader (Enspire, Perkin Elmer, Waltham, MA, USA) was used. At least six replicates for each sample were examined, and each experiment was independently performed at least three times. The percentage of inhibition of cell growth was calculated by the following Formula (1):

% growth = mean OD sample/mean OD control × 100 (1)

### 2.5. Wound Healing Assay

The effect of HEE on the migration potential of HepG2 and A549 cells was examined with the wound healing assay as described previously [24]. Briefly, cells were seeded in 6-well plates and, upon the formation of a confluent monolayer, a 10 µL pipette tip was used to generate a ‘wound’ on the monolayer by scratching it across the well. Loose and detached cells were washed away with PBS and cells were incubated with either DMSO (control) or HEE. Non-toxic HEE concentrations were used (20 or 30 μg/mL for A549 and 25 or 50 μg/mL for HepG2 cells) to prevent cell growth inhibition. Cells were monitored and photographed with a light microscope (Zeiss, Göttingen, Germany) and equipped with a digital camera, up until wound closure. Multiple photographs per time point were captured in three independent experiments. Photographs were analyzed with ImageJ software (NIH, Bethesda, MD, USA) and the average % wound area (% open image area) was calculated.

### 2.6. Chemical Characterization

#### 2.6.1. Sample Processing

For the determination of the total phenolic (TPC), flavonoid (TPC), soluble sugar (TSSC), soluble protein (TSPC), and pigment content, the dried HEE was resolubilized in methanol, whereas HAE was resolubilized in LC-MS grade water. Both reconstituted extracts were filtered through a 0.45 μm membrane filter (Sartorius Stedim biotech, Guttenberg, Germany). The formed solutions were aliquoted and stored at −20 °C protected from light until further use.

#### 2.6.2. Determination of Total Phenolic Content (TPC) and Total Flavonoid Content (TFC)

The TPC, of reconstituted solutions of HAE and HEE, was analyzed using a commercial polyphenolic quantification assay kit (Folin–Ciocalteu method) (KB03006, Bioquochem, Asturias, Spain) according to the manufacturer’s instructions. The TPC was determined based on the gallic acid calibration curve (linear range: 0–500 μg/mL, y = 0.004496x + 0.01785, R^2^ > 0.990). The results were expressed as μg of gallic acid equivalents/g of dry extract. The quantification of TFC was performed as it was previously reported with some modifications [25]. Briefly, 40 μL of each solution (HAE or HEE, respectively) was diluted with 120 μL of methanol and mixed with 20 μL of aluminum trichloride (10% aqueous solution) and 20 μL of sodium acetate (0.5 M aqueous solution). The resulting solutions were allowed to stand in the dark at RT for 40 min, and then the absorbance was monitored on a microplate reader (LT4500, Labtech, Heathfield, UK) at 415 nm. The TFC was determined based on the rutin calibration curve (linear range: 0–500 μg/mL, y = 0.0006949x + 0.04410, R^2^ > 0.993). The results were expressed as μg of rutin equivalents/g of dry extract.

#### 2.6.3. Determination of Total Condensed Tannins Content (TCTC)

The determination of TCTC was performed according to a previously published experimental protocol [26]. Briefly, 500 μL of either HAE or HEE reconstituted solution, was diluted with 500 μL of 70% acetone. Then, 3 mL of the *n*-butanol/hydrochloric acid (37%) mixture (95:5 % *v*/*v*) was added, and the resulting solutions were heated at 95 °C for approximately 60 min. Upon completion of the reaction, the solution mixture was allowed to cool at RT, mixed with ammonium iron (III), sulfate (NH_4_Fe(SO_4_)_2_) 2% (*w*/*v*), and heated for 2 h at 70 °C. Eventually, the absorbance of the cooled solution mixture was monitored on a microplate reader (LT4500, Labtech, Heathfield, UK) at 550 nm. The TCTC was determined based on a catechin calibration curve (linear range: 10–100 μg/mL, y = 001912x − 0.02036, R^2^ > 0.993). The results were expressed as μg of catechin equivalents/g of dry extract.

#### 2.6.4. Determination of Total Monoterpenoid Content (TMC)

The determination of TMC was performed by adopting a previously reported methodology [27]. Namely, 200 μL of either HAE or HEE reconstituted solution, was mixed thoroughly with 1.5 mL chloroform and allowed to stand for 3 min. Then, 100 μL of concentrated sulfuric acid were added and the suspensions were allowed in the dark for 2 h. The supernatant was decanted, the formed precipitant was taken up in 95% (*v*/*v*) methanol, and the absorbance was monitored on a microplate reader (LT4500, Labtech, Heathfield, UK) at 538 nm. The total monoterpenoid content was determined based on a linalool calibration curve (linear range: 0–60 μΜ, y = 0.005074x + 0.003620, R^2^ > 0.995). The results were expressed as μg of linalool equivalents/g of dry extract.

#### 2.6.5. Determination of Total Soluble Sugar Content (TSSC)

The determination of TSSC was performed as it was previously reported with some modifications [25]. Briefly, 150 μL of either HAE or HEE reconstituted solution was dehydrated by adding 150 μL of concentrated sulfuric acid, and the resulting solution mixture was shaken for 30 min at RT. Then, 30 μL of 5% phenol was added, and the final mixture was heated at 90 °C for 5 min. The absorbance of the cooled solutions was monitored on a microplate reader (LT4500, Labtech, Heathfield, UK) at 490 nm. The TSSC was determined based on the sucrose calibration curve (linear range: 0–100 nM, y = 0.01645 + 0.1578, R^2^ > 0.998). The results were expressed as nmol of mannose equivalents/g of dry extract.

#### 2.6.6. Determination of Total Soluble Protein Content (TSPC)

TSPC content was determined by utilizing the bicinchoninic acid (BCA) protein assay kit (Thermo Scientific, Waltham, MA, USA), according to the manufacturer’s instructions, and the absorbance was monitored at 562 nm using a microplate reader (LT4500, Labtech, Heathfield, UK). The TSPC was calculated based on a standard curve of bovine serum albumin (BSA) (linear range: 0–2 mg/mL, y = 0.6851x + 0.1345, R^2^ > 0.995). The results were expressed as mg of protein/g of dry extract.

#### 2.6.7. Determination of Pigments

The total content in chlorophyll-a and -b, lycopene, and β-carotene of either HAE or HEE reconstituted solutions, was determined as previously described [28]. The absorbance was measured sequentially at 453, 505, 645, and 663 nm using a microplate reader (LT4500, Labtech, UK), and the content was calculated using the following Equations (2)–(5):

Chlorophyll-a (mg/g of dry extract) = [((0.999 × A_663_) − (0.0989 × A_645_))/20] (2)

Chlorophyll-b (mg/g of dry extract) = [((1.77 × A_663_) − (0.328 × A_663_))/20] (3)

Lycopene (mg/g of dry extract) = [((−0.0458 × A_663_) + (0.204 × A_645_) + (0.372 × A_505_) − (0.0806 × A_453_))/20] (4)

β−carotene (mg/g of dry extract) = [((0.216 × A_663_) − (1.22 × A_645_) − (0.304 × A_505_) + (0.452 × A_453_))/20] (5)

The results were expressed as μg of pigment (chlorophyll-a or -b or lycopene or β-carotenoid)/g of dry extract.

#### 2.6.8. Determination of Volatiles

Chemical analysis of polar metabolites contained in an ethanolic sample of H. rhodopensis was performed as previously described [29]. Derivatization by silylation of the metabolites was initiated by the addition of methoxyamine hydrochloride followed by the addition of N-methyl-N-trimethylsilyl-trifluoroacetamide (MSTFA) to complete the reaction. The samples were analyzed by gas chromatography/mass spectrometry (GC/MS) on a Thermo Trace Ultra GC gas chromatography system with ISQ MS mass spectrometry and TriPlus RSH (Switzerland). The identification of metabolites and the analysis of mass spectra were performed using databases such as NIST11 and GOLM, while also for the identification of peaks, a comparison was made with standard substances.

#### 2.6.9. Preparation of Standards and Samples

Stock solutions of 4-hydroxybenzoic acid, protocatechuic acid, gallic acid, vanillic acid, syringic acid, *p*-coumaric acid, caffeic acid, ferulic acid, rosmarinic acid, chlorogenic acid, ellagic acid, 7-hydroxyflavanone, 4′-methoxyflavanone, apigenin-7-*O*-glucoside, isorhamnetin, quercetin-3-*O*-rhamnoside, quercetin-3-*O*-rutinoside (rutin), naringin, kaempferol-3-*O*-rutinoside, hyperoside, and myricetin-3-*O*-galactoside were prepared in methanol, luteolin-7-*O*-glucoside in acetonitrile/water mixture (1:1) and 2′-hydroxyflavanone, 5-methoxyflavanone and ipriflavone in methanol/acetonitrile mixture (1:1) at a concentration of 1000 ppm. Working standard solutions were made by diluting the individual standard stock solutions with ice-cold methanol. Both solutions of HEE and HAE were diluted with ice-cold methanol at a final concentration of 25 ppb. Each solution was kept in the dark and protected from light to minimize the autooxidation of polyphenols and pigments. In addition to this, both stock, standard, and sample solutions were stored at −20 °C before use. All prepared solutions were passed through 0.22 μm membrane filtered prior to UPLC-QqQ-ESI-MS/MS analysis.

#### 2.6.10. Liquid Chromatography (LC) Conditions

For the detection and quantification of the listed polyphenols, a Waters Acquity UPLC system (Waters Corp., Milford, MA, USA) equipped with an autosampler chamber, two pumps, and a degasser was used. The chromatographic separation was performed on an ACQUITY UPLC BEH C18 (100 × 2.1 mm, particle size: 1.7 μm) column (Waters Corp., Milford, MA, USA), heated at 30 °C and eluted as it was previously reported but with some modification [30]. Briefly, the mobile phase consisted of a solution of acetonitrile (eluent A) and formic acid 0.1% (*v*/*v*) (eluent B). A flow rate of 0.3 mL/min was used and the linear gradient conditions applied consisted of 5–100% A (0–4 min), 100–90% A (4.0–4.1 min), 90% A (4.1–5 min), 90–5% A (5–5.1 min), and 5% A (5.1–6 min). The injection volume was 10 μL and the autosampler temperature was set at 4 °C.

#### 2.6.11. MS/MS Conditions

For the MS/MS experiments, a Xevo tandem (triple) quadrable (QqQ) detector (TQD) mass spectrometer (Waters Corp., Milford, MA, USA) was operated in either positive or negative ionization mode (ESI^±^). Quantitative analysis was accomplished using selected multiple reaction monitoring (MRM) mode (Figures S1 and S2). The MRM conditions were optimized for each standard by MS manual tuning of each standard prior to sample analysis at a concentration of 1 ppm (Table S1). To acquire maximum signals, the optimized tuning parameters were as follows: capillary voltage: 3.0 kV; cone voltage: 36 V; source temperature: 150 °C; dissolution temperature: 500 °C; source disolving gas flow: 1000 L/h; and gas flow: 20 L/h. High-purity nitrogen gas was used as the drying and nebulizing gas, whereas ultrahigh-purity argon was used as a collision gas. The data acquisition and processing were performed on MassLynx software (version 4.1).

### 2.7. Determination of Antioxidant Activity

#### 2.7.1. ABTS^●+^ Assay

For the determination of the ability of the HAE and/or HEE to inhibit the cationic radical of ABTS^●+^, the ABTS^●+^ assay kit (KF01002, Bioquochem, Asturias, Spain) was used according to the manufacturer’s instructions. Gallic acid (0–35 μg/mL), Trolox (0–15 μg/mL), and ascorbic acid (0–7 μg/mL) were used as positive controls, whereas the potency of HAE and HEE were examined in a range of concentrations varying from 0 to 80 μg/mL. The results were calculated as % Radical cation inhibition according to Equation (6)

% Radical cation inhibition = [1 − (A*_f_*/A_0_)] × 100 (6)

where A*_f_* is the absorbance recorded at 517 nm, 5 min after the addition of samples, and A_0_ is the absorbance of the non-inhibited radical cation at 517 nm and expressed as the half-maximal inhibitory concentration (IC_50_) in μg/mL.

#### 2.7.2. DPPH^●^ Assay

For the determination of the ability of the HAE and HEE to inhibit the DPPH^●^ radical, the DPPH^●^ assay kit (KF01007, Bioquochem, Asturias, Spain) was used according to the manufacturer’s instructions. Gallic acid (0–3.5 μg/mL), Trolox (0–15 μg/mL), and ascorbic acid (0–70 μg/mL) were used as positive controls, whereas the potency of HAE and HEE were examined in a range of concentrations varying from 0 to 80 μg/mL. The results were calculated as % Radical cation inhibition according to Equation (6) where A*_f_* is the absorbance recorded at 715 nm, and A_0_ is the absorbance of the non-inhibited radical cation at 715 nm and expressed as the half-maximal inhibitory concentration (IC_50_) in μg/mL.

#### 2.7.3. FRAP Assay

For the determination of the ability of HAE and HEE to reduce the ferrous cationic species, the fast FRAP assay kit (KF01006, Bioquochem, Asturias, Spain) was utilized and performed according to the manufacturer’s recommendation. Gallic acid 0–3.5 μg/mL), Trolox (0–15 μg/mL), and ascorbic acid (0–70 μg/mL) were used as positive controls, whereas the potency of HAE and HEE were examined in a range of concentrations varying from 0 to 80 μg/mL. The results were calculated as mmols of Fe^2+^/g of dry extract and expressed as the half-maximal inhibitory concentration (IC_50_) in μg/mL.

#### 2.8. Statistical Analysis

Data are presented as mean ± SD and are representative of at least three independent experiments. Sigma Plot v.11 software (Systat Software Inc., San José, CA, USA) was used for statistical analyses and for generating the graphs unless stated otherwise. For statistical comparisons, Student’s *t*-test was used for comparing two groups, whereas, for multiple group comparisons, one-way ANOVA was performed. Differences between groups were considered significant when *p* < 0.05 (* *p* < 0.05, ** *p* < 0.01, *** *p* < 0.001). EC_50_ values (Efficient Concentration; the concentration that induces a 50% cell growth inhibition) were calculated from the respective dose-response curves by regression analysis using a four-parameter logistic curve with the Sigma Plot v. 11 Software v.10.

## 3. Results

### 3.1. Growth Inhibitory Activity of HAE and HEE against a Panel of Cancer Cell Lines

**Figure 1:**
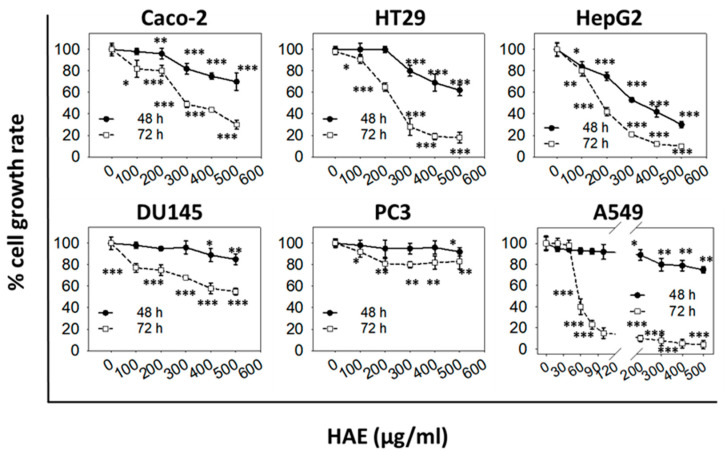
In vitro cancer cell growth inhibitory activity of HAE. Antiproliferative effect of increasing doses of HAE, for a 48 or 72 h treatment period, on a panel of six human cancer cell lines. The percentage (%) of cell growth was analyzed by the SRB assay. Data are representative of at least three independent experiments. Values represent means (n = 4) ± SD. Asterisks indicate a statistically significant difference in treated cells’ growth rate compared to control (* *p* < 0.05, ** *p* < 0.01, *** *p* < 0.001).

**Figure 2:**
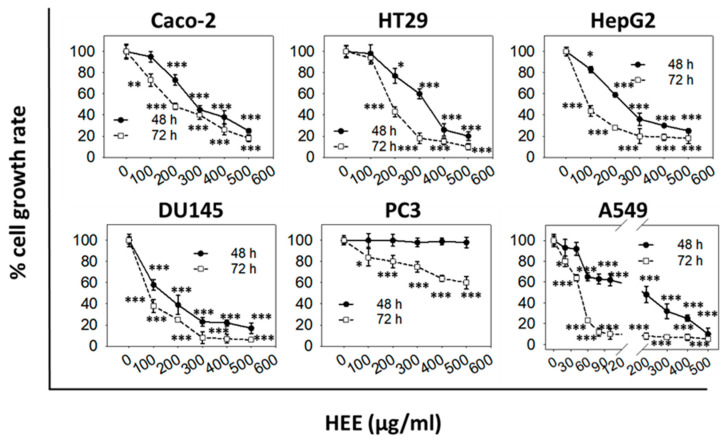
In vitro cancer cell growth inhibitory activity of HEE. Antiproliferative effect of increasing doses of HEE for a 48 or 72 h treatment period on a panel of six human cancer cell lines. The percentage (%) of cell growth was analyzed by the SRB assay. Data are representative of at least three independent experiments. Values represent means (n = 4) ± SD. Asterisks indicate a statistically significant difference in treated cells’ growth rate compared to control (* *p* < 0.05, ** *p* < 0.01, *** *p* < 0.001).

Both extracts exhibited a dose- and time-dependent growth inhibitory activity against a panel of six cancer cell lines (Figure 1 and Figure 2) with higher EC_50_ values for 48 h compared to 72 h post-treatment, respectively (Table 1 and Table 2).

Specifically, for HAE, the EC_50_ value at 48 h was determined to be 245 µg/mL for HepG2 cells whereas, for all other cell lines, it was not possible to be estimated (Table 1). However, at 72 h, post-treatment cell growth was inhibited by 50% for A549 (at 55 μg/mL), HepG2 (at 174 μg/mL), HT29 (at 218 μg/mL), and Caco-2 (at 236 μg/mL) cells. On the other hand, no EC_50_ values could be determined for both DU145 and PC3 cells (Table 1). Overall, it can be concluded that HAE moderately inhibits Caco-2, HT29, HepG2, and A549 growth, with A549 exhibiting the highest sensitivity to HAE, with Caco-2 as the lowest, at 72 h post-treatment (the higher the efficient concentration of HAE that causes a 50% decrease in cell growth, the less sensitive is the particular cell line to such treatment). Finally, HAE did not affect a significant extent the growth rate of either DU145 or PC3 cell lines at 48 and 72 h post-treatment. On the other hand, HEE exhibited a greater growth inhibitory effect against all cell lines tested. Notably, it was able to estimate the EC_50_ values for all cell lines except PC3, at 48 and 72 h post-treatment (Table 2). The highest EC_50_ value for HEE, at 72 hr, was observed in Caco-2 cells (168 ± 45 μg/mL, Table 2). Noteworthy, this value is lower by ≈30% as compared to the EC_50_ value calculated for a normal skin cell line, HaCaT cells, at 221 ± 53 μg/mL (Figure S4).

### 3.2. HEE Exerts Antimigratory Effects

**Figure 3:**
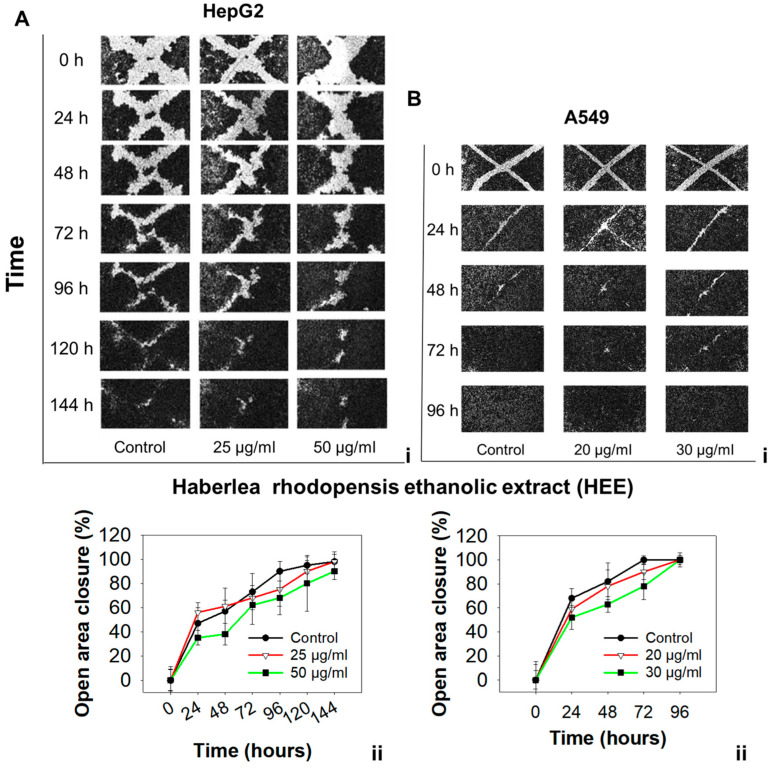
Effect of HEE on the migration of (**A(i)**) HepG2 and (**B(i)**) A549 cancer cells. The migration of cells was analyzed with the wound-healing assay and monitored with an optical microscope at the indicated time points. Quantification of the percentage of wound closure was analyzed by ImageJ for HepG2 (**A(ii)**) and A549 (**B(ii)**) cells, respectively. Data are presented as the mean ± SD of three independent experiments. Green asterisks indicate a statistically significant difference in treated (50 μg/mL) compared to control cells. Differences were considered statistically significant when *p* < 0.05 (Student’s *t*-test). No statistical difference was observed in control cells compared to cells treated with 25 or 20 μg/mL HΕΕ.

We sought to investigate whether HEE affects the migration of A549 or HepG2 cells, employing the wound-healing assay. Our results indicate that the open area (wound) in HEE-treated cells was filled in a concentration-dependent manner but at a slower pace compared to control cells in both cell lines (Figure 3A,B).

Specifically, for the A549 cell line, the mean ratio of open area closure in control cells after 24 h reached 68% compared to 59% for cells treated with 20 μg/mL HEE, or 52% for cells treated with 30 μg/mL HEE. At 48 h, the control group exhibited an 82% closure compared to 78% and 63% for cells treated with 20 or 30 μg/mL HEE, respectively. The wound in control cells was completely filled at 72 h, whereas complete closure took longer (96 h) for HEE-treated cells (Figure 3B). A similar effect was observed in HepG2 cells as well; an indicative delay in the progression of wound closure was observed at 96 h when control cells reached a 90% closure rate, compared to 75% or 68% for cells treated with either 25 or 50 μg/mL HEE. These differences were sustained for the entire 0–144 h time range examined (Figure 3A). It is worth noting that cells were treated with sub-toxic HEE concentrations that were determined based on our SRB results, to exclude HEE’s growth inhibitory effect as a potential mechanism of the observed delay of wound closure. Conclusively, considering the above reported results, HEE appears to exert antimigratory activity against both HepG2 and A549 cells.

### 3.3. Chemical Profiling of HEE—Optimization of UPLC and MS Conditions and Method Validation

Next, we focused on the chemical characterization of both HAE and HEE by performing UPLC-MS/MS analysis. Appropriate chromatographic conditions, combination of the mobile phase, elution mode, flow rate, and column used for the separation, were chosen in order to acquire the optimal (maximal) signal for all the analytes. For the determination of the optimum mobile phase, several combinations were applied, including methanol/water and acetonitrile/water in various percentages, however, none of them improved the shape and symmetry of the peaks. Acidification of water with 0.1% formic acid resulted in peaks with improved symmetry and shape. Additionally, the addition of formic acid facilitated the ionization of the compounds. Further improvement of all peaks was achieved by raising the column temperature to 30 °C. For the ionization of polyphenols, the electrospray ionization with either negative or positive (ESI^±^) mode was used.

The analytical method was validated according to the guidelines of the International Conference of Harmonization (ICH) [31]. Namely, parameters including, linearity, limit of detection (LOD), limit of quantification (LOQ), precision, and accuracy were determined. The generated calibration curves of the standards were plotted with a linear regression equation of peak areas versus various concentrations ranging from 0.65 to 510 ppb (Figure S3). All polyphenols demonstrated good linearity in the range of 0.65–505.6 ppb, whereas the correlation coefficients (R^2^) were > 0.999 for all of the analyzed standards (Table S2). Finally, we evaluated the reproducibility of the UPLC-QqQ-ESI-MS/MS method by means of determining the % of the recovery. For this purpose, the methanol solution of HEE was spiked with mixtures of standard solutions of various polyphenols. Spike samples were prepared in triplicates and the results were of at least six repetitions. The % recovery was calculated according to Equation (7):

%recovery = [(A − A_0_)/(A*_a_*)] × 100 (7)

where A is the final amount detected, A_0_ is the initial amount and A*_a_* is the added amount.

### 3.4. Determination of Phytochemicals, Nutraceuticals, and Pigments

Initially, we sought the determination of the total content of all major phytochemicals including phenolics, flavonoids, monoterpenoids, and condensed tannins in both extracts HAE and HEE (Table 3).

Overall, our results revealed that HEE contains significantly higher amounts of both flavonoids (249.89 ± 9.65 μg of rutin eq/g of dry extract) and phenolic compounds (359.50 ± 12.45 μg of gallic acid equivalents/g of dry extract) compared to HAE (107.89 ± 3.21 μg of rutin eq/g of dry extract and 121.45 ± 3.21 μg of gallic acid eq/g of dry extract, respectively) (Table 3). More particularly, HEE is enriched in protocatechuic acid (112.92 ± 0.28 μg/g of dry extract) and caffeic acid (59.90 ± 0.18 μg/g of dry extract). On the other hand, the evaluation of TSPC and TSSC in both fractions suggested that HAE contains higher amounts of soluble proteins (97.48 ± 2.21 mg of BSA eq/g of dry extract) and sugars (198.66 ± 8.41 nmols of sucrose eq/g of dry extract) compared to HEE (70.90 ± 1.82 mg of BSA eq/g of dry extract and 95.14 ± 8.13 nmols of sucrose eq/g of dry extract, respectively) (Table 3). Moreover, the detected levels of condensed tannins (85.94 ± 6.95 μg of catechin eq/g of dry extract) and total monoterpenoid (12.42 ± 3.21 μg of linalool eq/g of dry extract) appear to be significantly higher in the ethanolic extract compared the aqueous extract (47.65 ± 2.37 μg of catechin eq/g of dry extract and 1.65 ± 0.02 nmols of sucrose eq/g of dry extract, respectively) (Table 3). Finally, by utilizing a spectrophotometric approach, we were able to quantify the total concentration of pigments (chlorophyll-a, -b, β-carotene, and lycopene). As can be observed from Table 3, the concentration of chlorophyll-a and -b, in HEE, were in considerably higher proportion and especially chlorophyll-b which was determined to be at the highest concentration among the other pigments (489.5 ± 32.17 μg/g of dry extract) (Table 3). The same trend in the pigments profile appears in HAE; however, the detected levels were statistically lower compared to the HEE (Table 3).

### 3.5. Polyphenolics, Sugars, and Volatile Organic Acids

The content of polyphenolic compounds (including phenolic acids, flavanones, flavones, flavanols, and isoflavones) in the HAE and HEE was determined by UPLC-ESI-MS/MS. Peak identification was carried out by comparing the MRM transitions of the standard compounds. The chromatographic findings are expressed in μg/g of dry extract (Table 3). In HEE, protocatechuic acid (112.9 ± 0.28 μg/g of dry extract) and caffeic acid (59.9 ± 0.18 μg/g of dry extract) were determined to be at the highest concentration, among the other phenolic acids, whereas ipriflavone (92.40 ± 0.02 μg/g of dry extract) and quercetin-3-*O*-rutinoside (16.08 ± 0.01 μg/g of dry extract) were also at the highest quantity among all flavonoids (Table 3). On the other hand, lower levels of all analytes were detected in HAE compared to HEE. Interestingly, in HAE, caffeic and protocatechuic acids exist in almost the same quantity, and the levels of Myricetin-3-*O*-galactoside did not change significantly among the two extracts (Table 3).

Having observed that HEE exerts significantly stronger bioactivity compared to HAE, we proceeded to analyze by GC-MS the volatile organic acids and sugars of the ethanolic extract (Table S3). Results suggest that among them, the abundance of ribonic acid is 6.43-fold higher whereas that of sucrose is 109.77-fold higher when compared to the internal standard. Finally, HEE appears to be rich in *D*-(-)-Tagatofuranose, with its abundance being 8.90-fold higher than the internal standard.

### 3.6. Antioxidant Capacity of the HAE and HEE Fractions

**Figure 4:**
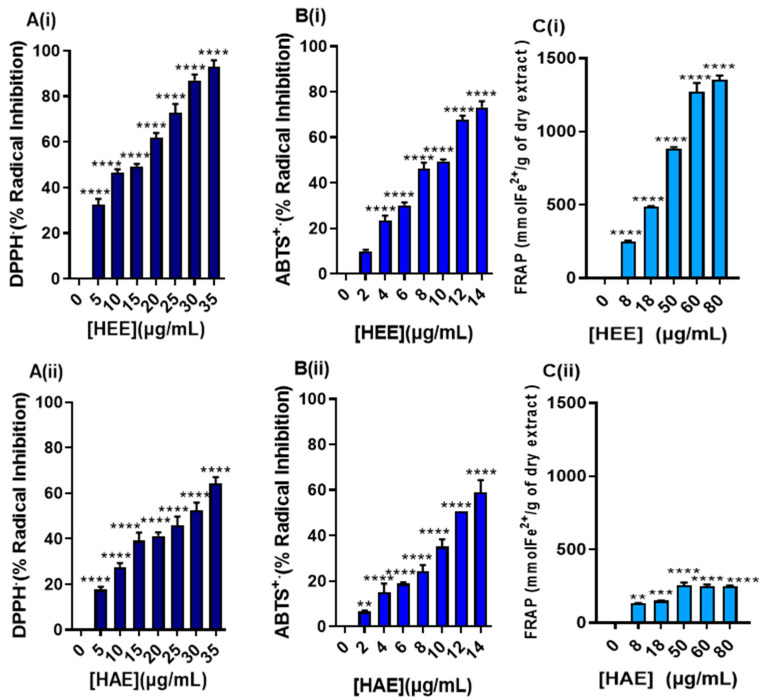
Antioxidant activity of HEE (**i**) and HAE (**ii**) as determined by (**A**) DPPH^●^, (**B**) ABTS^●+^, and (**C**) FRAP assays. Data are expressed as means ± SEM and are representative of three independent experiments. Statistical comparisons were conducted between the control (blank) and extract (in different concentrations). Asterisks ** denote statistical significance at *p* ≤ 0.01, *** at *p* ≤ 0.001, whereas **** at *p* ≤ 0.000.

Due to the high content of compounds with a known antioxidant capacity including polyphenols (phenolic acids and flavonoids), monoterpenoids, catechins, and pigments, we examined whether HEE exhibits antioxidant properties in a cell-free system. For this purpose, we monitored the ability of HEE to inhibit the cationic radical of ABTS^●+^ and DPPH. In addition, HEE’s ability to reduce ferrous cationic species was also evaluated by the FRAP assay. The antioxidant capacity of HAE was also examined under the same experimental conditions. Positive controls such as Trolox, ascorbic acid, and gallic acid were also used in various concentrations (Figure S5). The results revealed that HEE exhibited significant antioxidant activity in a concentration-depended manner (Figure 4A(i)–C(i)). HAE also exhibited antioxidant properties (Figure 4A(ii)–C(ii)) to a lesser extent though compared to HEE.

The IC_50_ values estimated by the ABTS, DPPH, and FRAP assays are presented in Table 4.

More specifically, in the ABTS^●+^ cationic radical inhibition assay, an improved antioxidant activity was noticed when the IC_50_ of HEE (8.2 ± 0.2 μg/mL) was compared to Trolox (11.7 ± 0.66 μg/mL) or gallic acid (IC_50_= 17.4 ± 1.2 μg/mL). However, this was not the case when HEE’s IC_50_ value was compared to ascorbic acid’s (IC_50_= 4.4 ± 0.16 μg/mL) (Table 4). In the case of the DPPH^●^ assay, HEE appears to act as a better radical inhibitor (IC_50_= 17.7 ± 1.2 μg/mL) compared to ascorbic acid only (IC_50_= 22.0 ± 1.2 μg/mL) (Table 4). Finally, we evaluated the ability of HEE to reduce ferrous cations (Fe^2+^) by a FRAP assay (Figure 4C). For this purpose, a range of HEE concentrations (0–80 μg/mL) were used while the results were compared with the concentrations of the positive standards previously used. Our results revealed that HEE was more potent compared to Trolox or ascorbic acid but less potent compared to gallic acid. Finally, it can be concluded that HAE also has an antioxidant potency, however, its marked IC_50_ values were higher compared to both HEE’s and the positive controls’ (for the DPPH^●^ and ABTS^●+^ assays).

## 4. Discussion

The use of various plant extracts as a source of medicinal agents (pharmaceuticals), remedies, cosmetics, and food additives continues to be an attractive alternative for the industry. For centuries, the local population has gained knowledge and experience in using various plant extracts in folk medicine, local kitchen (cuisine), and religious rituals. This tradition continues today when the use of the enormous local plant biodiversity is supported with extensive scientific research. Working together in RESBIOS, a Horizon 2020 project dedicated to incorporating the Responsible Research and Innovation (RRI) principles [32] in science, the groups from AgroΒioΙnstitute in Bulgaria and Democritus University of Thrace in Greece, found a scientific field of joint interest—the potential use of plant extracts of local interest in human cancer treatment. Moreover, we chose to focus our research on the locally endemic species *H. rhodopensis*. *H. rhodopensis* is a rare species, endemic to certain parts of Bulgaria and Greece. Fostering a responsible research approach in respecting bioconservation in our study, we utilized only extracts from biotechnologically obtained plants, since the species is protected. It is confirmed that in vitro-developed plants that are adapted in pots and grown under controlled conditions are highly similar to those collected from nature [21].

*H. rhodopensis* belongs to the small group of so-called resurrection plants known for the extreme desiccation tolerance of their vegetative parts. Being a subject of numerous extensive studies, it has been concluded that *H. rhodopensis* exerts significant antioxidant properties as other resurrection species do, to ensure desiccation tolerance. The significant antioxidant activity that *H. rhodopensis* extracts have, increased the interest in studies in medicinal and human well-being fields [6]. However, it is difficult to find any ethnobotanical-based evidence indicating the use of *H. rhodopensis* by the local population in the past. In the last 20–30 years though, the interest in various potential biological activities of these extracts raised enormously. Various extraction techniques and solvents have been used but as a rule, the studies on the biological activity of the extracts obtained were not accompanied by any chemical characterization of their content. Most studies so far have been performed on methanol extracts [3,20,33,34]. In general, aqueous and ethanol extracts are considered less toxic and more suitable for in vitro bioactivity studies. In the present study, we used aqueous and ethanol extracts from air-dried leaves of *H. rhodopensis* plants in order to perform a comparative study on their antiproliferative potential against a panel of cancer cell lines and further focused on the ethanolic extract of the plant that demonstrated stronger bioactivity.

The comparison of aqueous (HAE) and ethanol (HEE) extracts showed that both of them exhibited a dose- and time-dependent growth inhibitory activity. However, HAE induced weaker antiproliferative effects compared to HEE (Figure 1 and Figure 2, Table 1 and Table 2). Similar results reporting weak cytotoxicity for the aqueous extracts have been described earlier [15] and in this respect, it was decided to focus on HEE. Methanol extracts promoted H_2_O_2_-induced apoptosis in prostate cancer (PC3) cells and exerted a protective effect against the non-cancerous (HEK293) cell line under the same experimental conditions [11].

In our study, the antiproliferative activity of the extracts was assessed using a platform of six different human cancer cell lines. We found that the different types of cancer cells had differential sensitivity to either HAE or HΕE with the more sensitive being the androgen-independent prostate (DU145), the hepatocellular carcinoma (HepG2), and the non-small cell lung adenocarcinoma (A459) cells. HEE also significantly affected the growth of the colon adenocarcinoma (Caco-2 and HT29) cell lines whereas it did not appear to have any effect on the growth of prostate (PC3 and DU145) cancer cells. The differential bioactivity of HEE, on the two prostate cancer cell lines tested, could be associated with their differences in expressing prostate-specific antigen (PSA) [35]. Moreover, DU145 cells have moderate metastatic potential compared to PC3 cells which have a high metastatic potential [36]. Noteworthy, contrary to cancer cells, normal cells were not equally sensitive to HEE as shown in Figure S4. An EC_50_ value of 221 ± 53 μg/mL was estimated for HaCaT keratinocytes at 72 h, while the highest EC_50_ value observed for the cancer cells lines examined, was 168 ± 45 μg/mL for Caco-2 cells. The lower ≈30% EC_50_ value in cancer cells as compared to normal cells, indicates that cancer cells, under the experimental conditions employed, are more susceptible to the growth inhibitory effects exerted by HEE compared to the normal cell line tested. Finally, a significant antimigratory potential of HEE was observed against HepG2 and A549 cells. To our knowledge, this is the first study describing the antiproliferative and antimigratory effects of *H. rhodopensis* extracts that may be of significant value for further exploring their anticancer potential.

To this end, our data prompted us to further characterize the chemical composition of both HAE and HEE. HEE was found to contain both flavonoids and phenolic compounds in abundance with the latter being more prominent. On the contrary, the levels of monoterpenoids and condensed tannins were present at low levels. Other significant molecules included soluble proteins and sugars both of which were present at high and low levels, respectively. In addition, the HEE extract contained high amounts of pigments (chlorophyll-a, -b, lycopene, and β-carotene) with chlorophyll-a being present at the highest concentration levels among the other pigments. On the other hand, analysis of HAE indicates the presence of polyphenolic compounds and pigments, amounting to much less when compared to HEE. In addition to this, it was observed that HAE contains a higher amount of soluble proteins and sugars when compared to HEE. The results of our study are in accordance with the main type of bioactive molecules that have been identified in various *H. rhodopensis* extracts, as summarized in Georgiev et al. [2], and include flavonoids, phenolic acids, fatty acids, phytosterols, polysaccharides, carotenoids, and other lipid-soluble constituents.

Analysis of the polyphenolic profile (including phenolic acids, flavanones, flavones, flavanols, and isoflavones) of the HEE extract revealed that protocatechuic acid and caffeic acid were the most abundant among the phenolic acids whereas ipriflavone and quercetin-3-*O*-rutinoside were also detected at higher concentrations amongst the flavonoids. We also found other noteworthy phenolic acids in abundance including ellagic acid, gallic acid, ferulic acid, syringic acid, rosmarinic acid, 4-hydroxybenzoic acid, and chlorogenic acid. In the case of flavonoids, quercetin-3-*O*-rutinoside, kaempherol-3-*O*-rutinoside, isorhamnetin, luteolin-7-*O*-glucoside, hyperoside, 7 hydroxyflavanone, apigenin-7-*O*-glucoside, naringin, 2′-hydroxyflavone, 4′-methoxyflavone, and quercetin-3-*O*-rhamnoside were all shown to be present. A large number of flavonoids and phenolics were identified earlier by HPLC analysis in a 70% ethanol extract of *H. rhodopensis* (collected from their natural habitat), prepared by conventional heat reflux extraction, where sinapic, ferulic, and *p*-coumaric acids were the most abundant amongst the phenolic acids while luteolin followed by hesperidin were the most predominant among the flavonoids [37]. In another study, HPLC analysis on ultrasound-based *H. rhodopensis* ethanol extract revealed a large number of phenolic acids and flavonoids to be present, with the most abundant phenolic acids being ferulic and sinapic acids, while the major flavonoids were luteolin, hesperidin, and kaempferol [19]. Sinapic acid was not included in our authentic standards in this study and so there is no information about its content in the HEE fraction however, no traceable p-coumaric acid levels were detected. Syringic acid was the most abundant among the five free phenolic acids when GC-MS analyses were performed with methanol extracts [20]. It is obvious, that the differences in the nomenclature of metabolites identified are related to the origin of the plant material used and the extraction methodologies followed. Phenolic compounds, on the other hand, are well known to play a significant role in the orchestrated response of plants under stress conditions and their recruitment, at high levels, is well documented in important physiological functions of the resurrection plants [38].

As it was anticipated, based on the high phenolic and flavonoid content of the HEE, a remarkable antioxidant capacity was shown as measured by three independent cell free-based methodologies including the DPPH^●^ radical scavenging activity, ABTS^●+^ cation decolorization activity, and FRAP assays. Moreover, such activity was comparable to other potent antioxidants like Trolox as well as gallic and ascorbic acids. High in vitro antioxidant activity for *H. rhodopensis* extracts have also been reported by other studies using various assays [2]. On the other hand, the antioxidant evaluation of HAE demonstrated some radical scavenging potency. However, the marked capacity was considerably weaker compared to the one noticed for HEE. This difference can be attributed to the low content of ROS scavenger molecules (e.g., low content in polyphenolic acid, flavanones chlorophylls, etc.). The observed antioxidant activity of HEE is promising and indicative of potential cytoprotective properties. *H. rhodopensis* as a source of antioxidant compounds should be further explored by utilizing physiologically relevant cell-based models and in vivo studies to demonstrate its possible health-promoting effects as well as potential biomedical applications.

## 5. Conclusions

The purpose of the present study was to analyze the antiproliferative potential of aqueous and ethanolic extracts derived from leaves of in vitro propagated *H. rhodopensis* plants and further characterize their chemical constitution, exploring any potential health-promoting and/or disease-preventing properties that would be of pharmaceutical interest. The ethanolic extract (HEE) was more potent than the aqueous one (HAE) in inducing inhibition of cancer cell proliferation as observed after screening against a panel of six human cancer cell lines. The results of our study show that HEE had an antiproliferative effect against the majority of cancer cell lines tested in a concentration- and time-dependent manner. We have also investigated HEE’s antimigratory potential as a mechanism of anticancer action. HEE was found to delay wound closure, therefore inhibit migration in both HepG2 and A549 cells. Chemical characterization revealed that HEE contains higher levels of flavonoids, phenolic compounds, monoterpenoids, condensed tannins, and pigments compared to HAE. On the other hand, HAE was found to be enriched in soluble proteins and sugars to a much higher extent compared to HEE. Furthermore, HEE was found to exert not only more significant antiproliferative activity against cancer cells, but to possess stronger antioxidant properties as well, an observation that could be attributed to the higher HEE content in phenolic compounds (flavonoids, monoterpenoids, etc.), compared to HAE. Future studies should aim at identifying the chemical constituents responsible for the bioactivities observed, and focus on investigating HEE’s anticancer effects, in in vivo preclinical cancer models.

## Figures and Tables

**Table 1:** EC_50_ values (efficient concentration that causes a 50% decrease in cell growth) of HAE (for a 48 or 72 h treatment period) against a panel of six human cancer cell lines. Data are representative of at least four independent experiments and are presented as means ± SD. Asterisks represent the statistical *p*-value (*** *p* < 0.001).

	Caco-2 (μg/mL)	HT29 (μg/mL)	DU145 (μg/mL)	PC3 (μg/mL)	HepG2 (μg/mL)	A549 (μg/mL)
**EC_50_ (48 h)**	n.d.	n.d.	n.d.	n.d.	245 ± 15 ***	n.d.
**EC_50_ (72 h)**	236 ± 10 ***	218 ± 8 ***	n.d.	n.d.	174 ± 2 ***	55 ± 3 ***

n.d.: not detected.

**Table 2:** EC_50_ values (efficient concentration that causes a 50% decrease in cell growth) of HEE for a 48 or 72 h treatment period against a panel of six human cancer cell lines. Data are representative of at least four independent experiments and are presented as means ± SD. Asterisks represent the statistical *p*-value (* *p* < 0.05, ** *p* < 0.01, *** *p* < 0.001).

	Caco-2 (μg/mL)	HT29 (μg/mL)	DU145 (μg/mL)	PC3 (μg/mL)	HepG2 (μg/mL)	A549 (μg/mL)
**EC_50_ (48 h)**	248 ± 28 **	329 ± 92 ***	115 ± 7 **	n.d.	197 ± 21 **	150 ± 57 *
**EC_50_ (72 h)**	168 ± 45 *	176 ± 5 ***	67 ± 16 *	n.d.	81 ± 3 ***	46 ± 3 **

n.d.: not detected.

**Table 3:** Composition of nutraceuticals and phytochemicals including total soluble sugar content (TSSC), total soluble protein content (TSPC), pigments (chlorophylls-a, -b, lycopene, and β-carotene), total phenolic content (TPC), and total flavonoid content (TFC), as well as individual phenolic acids. Flavonoids all of which were contained in the HAE and HEE extracts.

	HAE	HEE	Expression Units
**Total Phenolic Content (TPC)**	121.45 ± 3.21	359.50 ± 12.45 ****	μg of gallic acid eq/g of dry extract
**Phenolic acids**			
4-hydroxybenzoic acid	4.45 ± 0.14	1.86 ± 0.03 ****	μg/g of dry extract
Protocatechuic acid	26.97 ± 1.11	112.92 ± 0.28 ****	
Gallic acid	1.43 ± 0.21	10.04 ± 0.01 ****	
Vanillic acid	0.47 ± 0.02	11.48 ± 1.11 ****	
Syringic acid	n.d.	3.67 ± 0.010	
*p*-coumaric acid	2.120 ± 0.012	n.d.	
Caffeic acid	28.36 ± 1.27	59.90 ± 0.18 ****	
Ferulic acid	0.430 ± 0.001	5.60 ± 0.04 ****	
Rosmarinic acid	n.d.	2.51 ± 0.03	
Chlorogenic acid	n.d.	0.71 ± 0.01	
Ellagic acid	2.21 ± 0.01	18.400 ± 0.013 ****	
**Total Flavonoid Content (TFC)**	107.89 ± 3.21	249.89 ± 9.65 ****	μg of rutin eq/g of dry extract
**Flavonoids**			
2′-hydroxyflavanone	0.58 ± 0.02	1.730 ± 0.001 ***	μg/g of dry extract
7-hydroxyflavanone	1.88 ± 0.43	7.060 ± 0.021 ****	
4′-methoxyflavanone	n.d.	1.280 ± 0.011	
5-methoxyflavanone	n.d.	8.090 ± 0.019	
Apigenin-7-*O*-glucoside	1.32 ±0.13	4.36 ± 0.03 ***	
Luteolin-7-*O*-glucoside	2.26 ±0.21	8.56 ± 0.02 ****	
Isorhamnetin	4.65 ±0.32	8.70 ± 0.02 ****	
Quercetin-3-*O*-rhamnoside	n.d.	0.250 ± 0.001	
Quercetin-3-*O*-rutinoside	5.47 ± 0.25	16.080 ± 0.013 ****	
Hyperoside	3.45 ± 0.67	8.47 ± 0.01 ****	
Myricetin-3-*O*-galactoside	25.89 ± 1.18	24.12 ± 0.01	
Kaempferol-3-*O*-rutinoside	4.56 ± 0.31	15.620 ± 0.011 ****	
Ipriflavone	32.16 ± 1.44	92.40 ± 0.02 ****	
Naringin	n.d.	3.710 ± 0.011	
**Condensed Tannins Content**	47.65 ± 2.37	85.94 ± 6.95 ****	μg of catechin eq/g of dry extract
**Total mono-Terpenoid Content**	1.65 ± 0.02	12.42 ± 3.21 ****	μg of linalool eq/g of dry extract
**Total Soluble Protein Content (TSPC)**	97.48 ± 2.21	70.90 ± 1.82 ****	mg of BSA eq/g of dry extract
**Total Soluble Sugar Content (TSSC)**	198.66 ± 8.41	95.14 ± 8.13 ****	nmols of sucrose eq/g of dry extract
**Pigments**			
Chlorophyll-a	68.24 ± 3.21	156.80 ± 7.47 ****	μg of pigment/g of dry extract
Chlorophyll-b	93.17 ± 8.65	489.50 ± 32.17 ****	
*β*-Carotene	4.21 ± 1.27	81.20 ± 5.21 ****	
Lycopene	2.33 ± 0.48	22.00 ± 1.12 ****	

Values are the means of three independent experiments ± SD. Asterisks (****) denote statistical significance at *p* < 0.0001, whereas ***, denote significance at *p* < 0.001. n.d. not detected (values below LOD and LOQ limits).

**Table 4:** Antioxidant activity of HAE and HEE as determined by the DPPH^●^, ABTS, and FRAP assays.

Sample	DPPH^●^	ABTS^●+^	FRAP
IC_50_ (μg/mL)			
Gallic acid	1.8 ± 0.01	17.4 ± 1.2	14.79 ± 0.89
Ascorbic acid	22.0 ± 1.2	4.4 ± 0.16	39.42 ± 1.21
Trolox	7.6 ± 0.3	11.7 ± 0.66	41.45 ± 0.26
HEE	17.7 ± 1.2 ****	8.2 ± 0.2 ***	24.28 ± 1.32 ***
HEA	28.4 ± 0.4	11.8 ± 0.7	37.12 ± 1.11

Data are represented as means ± SEM from three independent experiments. Each assay was conducted in triplicates. Trolox, ascorbic, and gallic acids were used as positive controls. Asterisks indicate statistical significance (***) at *p* ≤ 0.001, whereas **** at *p* ≤ 0.0001.

## Data Availability

All data are contained within the article and Supplementary Materials.

## Supplementary Materials

The following supporting information can be downloaded at: https://www.mdpi.com/article/10.3390/antiox11122305/s1, Table S1: The optimal conditions for Multiple Reaction Monitoring (MRM) transitions of phenolic acid and flavonoids; Figure S1: The chromatograms of the phenolic acid standards in the MRM mode, ionized in the negative ESI; Figure S2: The chromatograms of the flavonoid standards in the MRM mode, ionized on both positive and negative ESI; Figure S3: Calibration curve of polyphenolic acids in a range of concentrations (0–500 ppb for phenolic acids and 1.95–250 ppb for flavonoids); Table S2: The LOD, LOQ, linearity, precision, and accuracy results for the screened of phenolic acids. Figure S4: Antiproliferative effect of increasing doses of HEE and EC_50_ value at 72 h against HaCaT cells. Data are representative of at least three independent experiments and are presented as means ± SD. Asterisks indicate statistical significance in treated cells’ growth rate compared to control (Student’s *t*-test, * *p* < 0.05, ** *p* < 0.01, *** *p* < 0.001). Table S3: GC–MS-based metabolite profiling of HEA (Haberlea rhodopensis ethanolic extract). Quantification of the detected metabolites was assessed based on the relative response compared to internal standard adonitol and expressed as relative abundance. Figure S5: Antioxidant capacity of the positive controls; Trolox, ascorbic acid, and gallic acid as determined by (**A**) DPPH^●^, (**B**) ABTS^●+^, and (**C**) FRAP assay. Data are expressed as means ± SEM and are representative of three independent experiments.
