# Supplementary material for: Chemical Profiling, Antiproliferative and Antimigratory Capacity of Haberlea rhodopensis Extracts in an In Vitro Platform of Various Human Cancer Cell Lines
Source: Antioxidants (Basel). 2022 Nov 22;11(12):2305. doi: 10.3390/antiox11122305 (PMC9774357; doi:10.3390/antiox11122305)
Supplement: Supplementary file 1 [file antioxidants-11-02305-s001.zip › antioxidants-2039186-supplementary.pdf]

Supplementary Materials

Table S1. The optimal conditions for Multiple Reaction Monitoring (MRM) transitions of phenolic acid and flavonoids.

Compound	Formula	MW	ESI	$[M-H]^{-/+}$ (m/z)	MS fragments (m/z)	Cone voltage (V)	Collision energy (eV)	Retention time (min)
4-hydroxybenzoic acid	$C_7H_6O_3$	138.12	negative	136.95	65.0	23	25	1.88±0.01
					93.0	23	13	
Protocatechuic acid	$C_7H_6O_4$	154.12	negative	152.95	108.95	25	13	1.64±0.01
Gallic acid	$C_7H_6O_5$	170.12	negative	168.95	78.98	23	22	1.37±0.02
					124.95	23	15	
Vanillic acid	$C_8H_8O_4$	168.14	negative	167.0	92.2	22	20	2.23±0.02
					119.9	22	15	
Syringic acid	$C_9H_{10}O_5$	198.17	negative	197.0	122.95	27	23	1.93±0.02
					182.0	27	13	
p-coumaric acid	$C_9H_8O_3$	164.16	negative	163.0	119.0	15	13	2.13±0.01
Caffeic acid	$C_9H_8O_4$	180.16	negative	178.95	134.95	25	13	1.89±0.03
Ferulic acid	$C_{10}H_{10}O_4$	194.18	negative	192.95	134.0	26	25	2.20±0.01
					178.0	26	12	
Rosmarinic acid	$C_{18}H_{16}O$	360.32	negative	359.2	161.0	10	15	2.26±0.01
					197.0	10	15	
Chlorogenic acid	$C_{16}H_{18}O_9$	354.31	negative	353.1	84.0	22	44	1.70±0.01
					191.02	22	14	
Ellagic acid	$C_{14}H_6O_8$	302.19	negative	301	145.0	35	34	2.00±0.01
					173.0	35	36	
2′-hydroxyflavanone	$C_{15}H_{12}O_3$	240.27	negative	239	119.3	40	25	3.42±0.02
					93.1	40	16	
7-hydroxyflavanone	$C_{15}H_{12}O_3$	240.27	negative	239.05	135.2	41	25	3.18±0.02
					91.15	41	23	
4′-methoxyflavanone	$C_{16}H_{14}O_3$	254.29	positive	255.15	240	31	17	3.78±0.01
					161.3	31	22	
5-methoxyflavanone	$C_{16}H_{14}O_3$	254.29	positive	255.15	151.3	34	22	3.49±0.01
Apigenin-7-O-glucoside	$C_{21}H_{20}O_{10}$	432.38	negative	431.15	268.35	35	22	2.16±0.02
Luteolin-7-O-glucoside	$C_{21}H_{20}O_{11}$	448.38	positive	449.15	287.1	34	31	2.01±0.01
Isorhamnetin	$C_{16}H_{12}O_7$	316.28	negative	315	151.0	43	30	2.83±0.03
					300.2	43	20	
Quercetin-3-O-rhamnoside	$C_{21}H_{20}O_{11}$	448.38	negative	447.01	271	43	47	2.27±0.01
					300	43	28	
Quercetin-3-O-rutinoside	$C_{27}H_{30}O_{16}$	610.53	negative	609.1	300	47	39	1.92±0.01
					271	47	65	
Hyperoside	$C_{21}H_{20}O_{12}$	464.38	negative	463.3	300	47	24	1.99±0.02
					271.15	47	44	
Myricetin-3-galactoside	$C_{21}H_{20}O_{13}$	480.38	negative	479.05	271.1	48	39	1.87±0.01
					287.1	48	44	
Kaempferol-3-O-rhamnoside	$C_{21}H_{20}O_{10}$	432.39	negative	431.05	255.3	45	42	2.27±0.01
					284.2	45	28	
Ipriflavone	$C_{18}H_{16}O_3$	280.33	positive	281.3	240	40	19	4.17±0.03
Naringin	$C_{22}H_{32}O_{14}$	580.54	negative	579.15	271.1	45	33	2.21±0.01
					151.5	45	40	

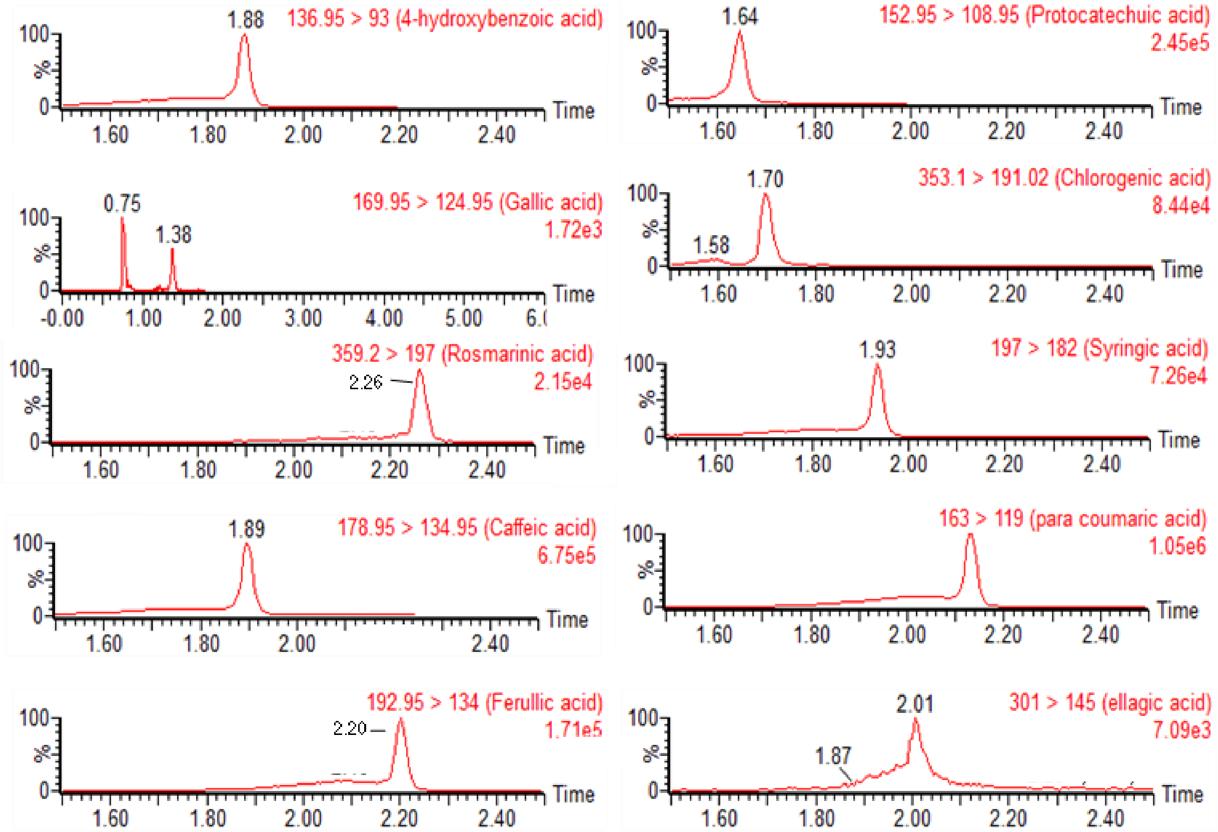

Figure S1. The chromatograms of the phenolic acid standards in the MRM mode, ionized in the negative ESI. The quantification signal is denoted into the spectra.

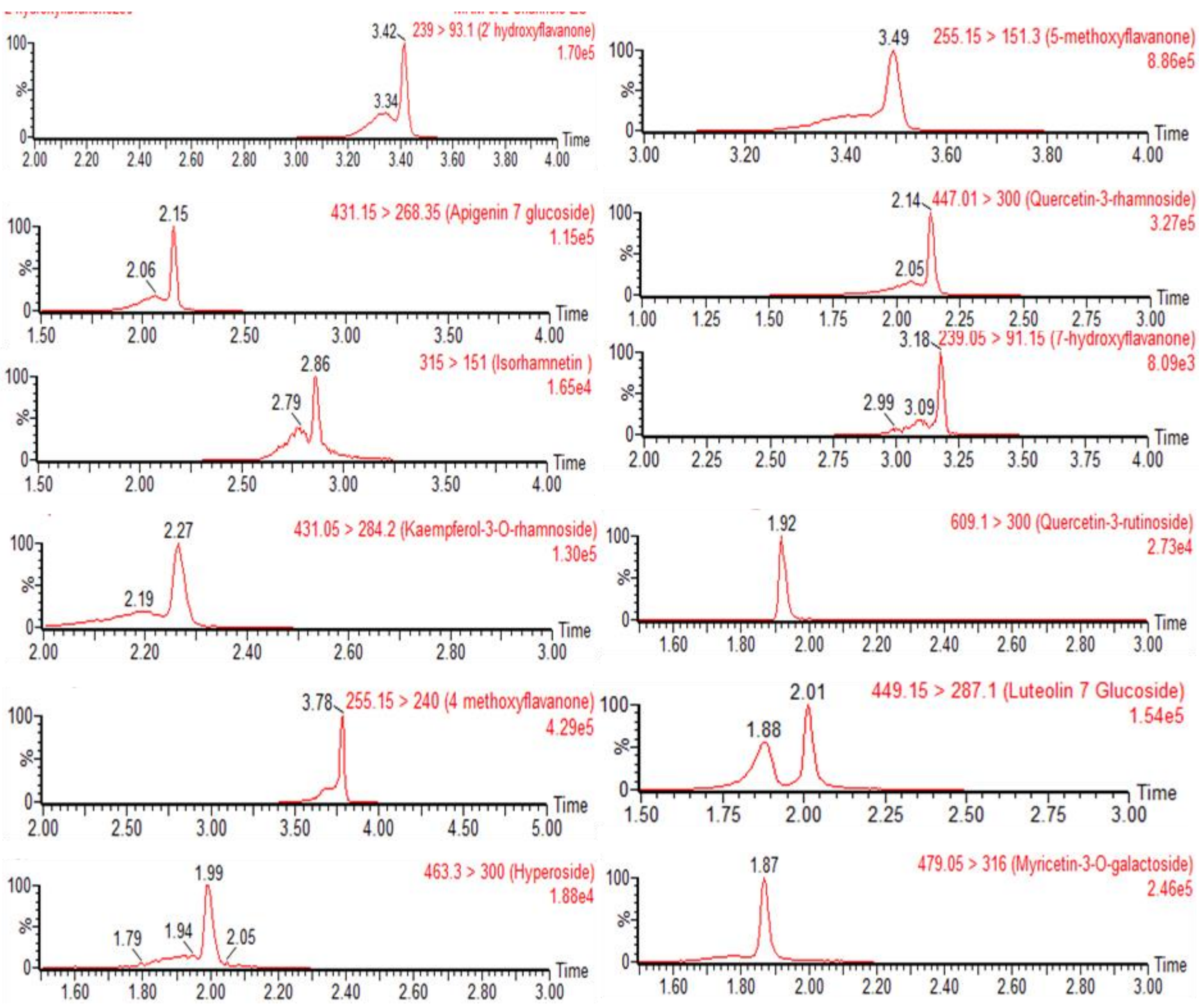

Figure S2. The chromatograms of the flavonoid standards in the MRM mode, ionized on both positive and negative ESI. The quantification signal is denoted into the spectra.

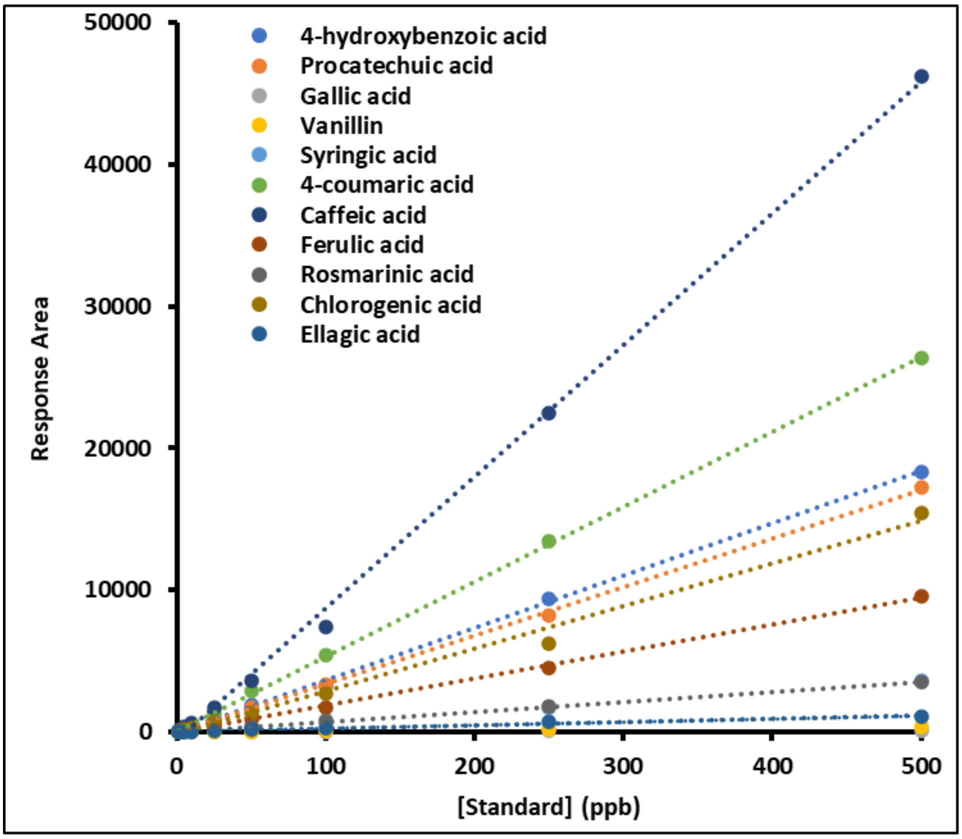
Response Area
[Standard] (ppb)
0
10000
20000
30000
40000
50000
0
100
200
300
400
500
4-hydroxybenzoic acid
Procatechuic acid
Gallic acid
Vanillin
Syringic acid
4-coumaric acid
Caffeic acid
Ferulic acid
Rosmarinic acid
Chlorogenic acid
Ellagic acid

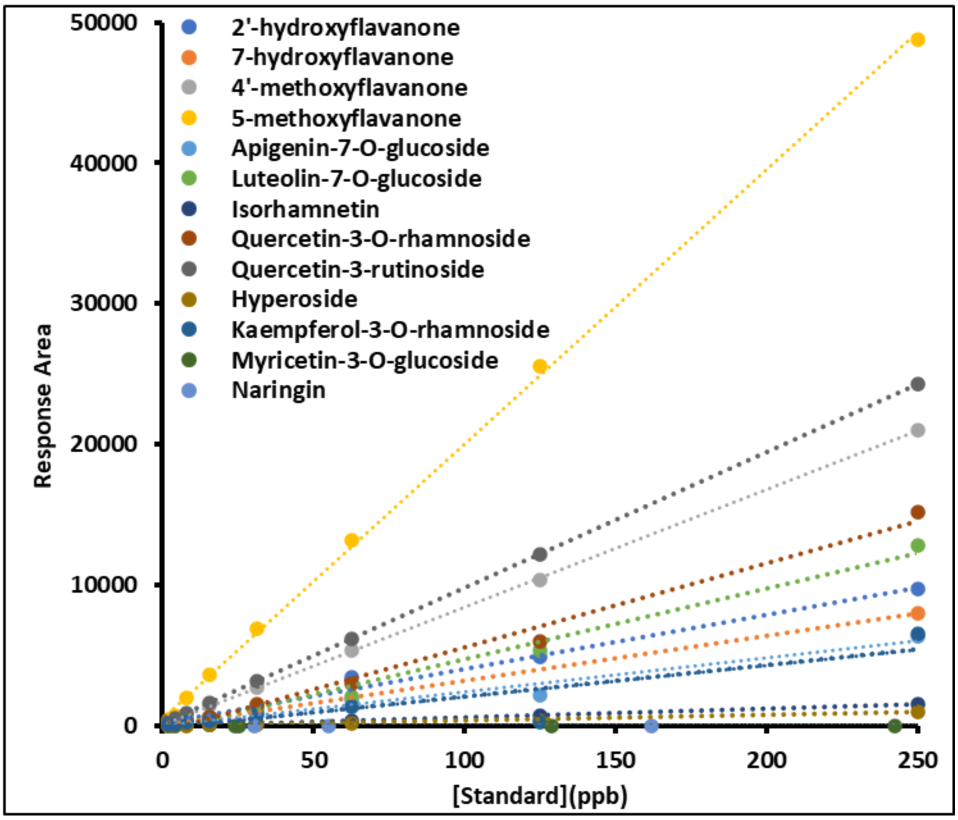
Response Area
[Standard](ppb)
0
10000
20000
30000
40000
50000
0
50
100
150
200
250
2'-hydroxyflavanone
7-hydroxyflavanone
4'-methoxyflavanone
5-methoxyflavanone
Apigenin-7-O-glucoside
Luteolin-7-O-glucoside
Isorhamnetin
Quercetin-3-O-rhamnoside
Quercetin-3-rutinoside
Hyperoside
Kaempferol-3-O-rhamnoside
Myricetin-3-O-glucoside
Naringin

Figure S3: Calibration curve of polyphenolic acids in a range of concentrations (0-500 ppb for phenolic acids and 1.95-250 ppb for flavonoids)

Table S2. The LOD, LOQ, linearity, precision and accuracy results for the screened of phenolic acids.

Compound	Linear range (ppb)	LOD (ppb)	LOQ (ppb)	Calibration equation[a]	Correlation coefficient (r^2)	%RSD		%REC[d]
						(intra-day)[b]	(inter-day)[c]	
**4-hydroxybenzoic acid**	**3.01-499.50**	**3.01**	**14.20**	**y=36.87x-62.07**	**0.9991**	1.1	2.28	102.9
Protocatechuic acid	0.66-504.50	0.66	14.70	y=34.24x-69.4	0.9995	1.2	4.31	99.8
Gallic acid	53.20-513.20	53.20	105.20	y=0.67x-1.5	0.9996	1.03	2.65	95.5
Syringic acid	2.01-501.60	2.01	2.86	y=7.28x-2.7	0.9996	2.02	4.10	93.7
p-coumaric acid	0.65-497.30	0.65	1.55	y=52.84x+36.9	0.9997	0.5	1.95	99.7
Caffeic acid	1.21-500	1.21	1.25	y=92.95x-344.4	0.9995	1.37	2.1	97.2
Ferulic acid	2.10-505.60	2.10	12. 17	y=19.02x-68.4	0.9992	4.2	2.53	100.5
Rosmarinic acid	2.32-499.50	2.32	2.56	y=7.03x+12.34	0.9996	0.78	3.0	101.2
Chlorogenic acid	3.48-495.60	3.48	4.76	y=25.02x+60.3	0.9991	0.98	1.99	94.5
Ellagic acid	5.53-499.10	5.53	75.60	y=2.18x+7.4	0.9995	0.57	4.6	95.4
2′-hydroxyflavanone	19.50-250.00	19.50	20.12	y=38.69x+22.5	0.9998	0.65	4.2	89.9
7-hydroxyflavanone	1.97-249.90	1.97	2.21	y=51.17x-73.6	1	0.69	4.2	95.5
4′-methoxyflavanone	2.21-250.00	2.21	3.89	y=83.54x+60.3	0.9999	0.47	4.3	92.2
5-methoxyflavanone	6.47-248.50	6.47	8.52	y=195.14x-493.9	0.9992	0.58	1.89	98.7
Apigenin-7-*O*-glucoside	1.87-125.30	1.87	4.42	y=6.17x+3.8	0.9998	1.12	3.02	96.5
Luteolin-7-*O*-glucoside	2.21-250.10	2.21	2.22	y=51.52x-89.9	0.9998	1.32	2.21	96.6
Isorhamnetin	14.01-251.1	14.01	2.31	y=6.08x-15.4	0.9992	1.54	1.87	89.9
Quercetin-3-*O*-rhamnoside	1.02-250.60	1.02	4.21	y=60.83x-38.6	0.9999	1.7	1.94	92.1
Quercetin-3-*O*-rutinoside	1.40-251.30	1.40	4.32	y=97.74x+109.7	0.9999	1.01	2.21	94.7
Hyperoside	6.32-249.90	6.32	3.21	y=3.97x+0.5	0.9998	0.7	2.45	94.3
Myricetin-3-galactoside	0.85-251.20	0.85	2.12	y=26.38x-31.8	0.9997	1.3	3.02	91.9
Kaempferol-3-*O*-rutinoside	0.76-250.00	0.76	1.21	y=25.73x+73.7	0.9997	1.35	1.98	97.7
Ipriflavone	109.90-250.00	109.90	130.21	y=0.62x+2.2	0.9994	1.32	3.05	90.9
Naringin	3.01-250.60	3.01	1.21	y=22.88x-43.3	0.9997	2.7	4.32	101.0

[a]Chromatographic peak area (y) as a function of ppb concentration (x). [b]Values are means of intra-day assays (*n*=6). [c] Values are means of inter-day assays (*n*=6). [d] (*n*=3).

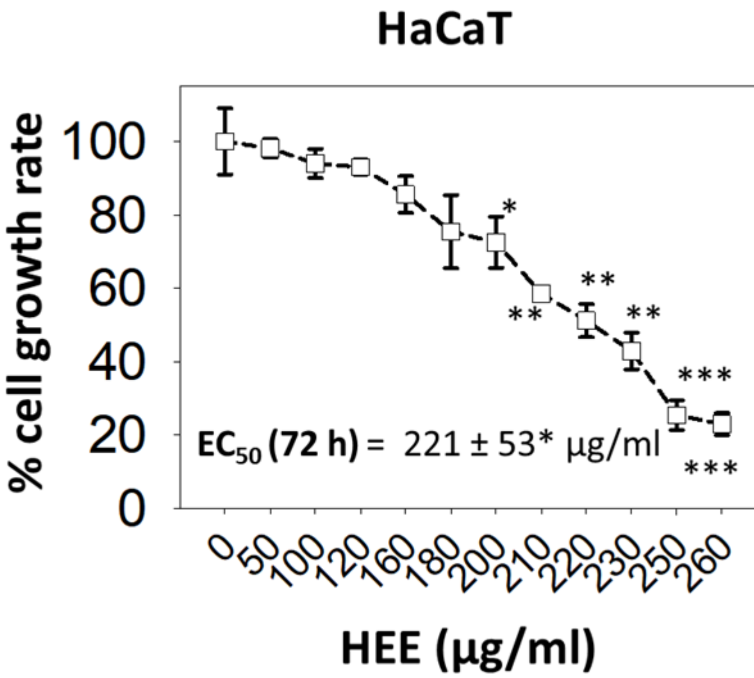

Figure S4. Antiproliferative effect of increasing doses of HEE and EC_{50} value at 72 hr against HaCaT cells. Data are representative of at least three independent experiments and are presented as means ± SD. Asterisks indicate statistical significance in treated cells growth rate compared to control (Student's t-test, *p<0.05, **p<0.01, ***p<0.001).

Table S3. GC–MS based metabolite profiling of HEA (Haberlea rhodopensis ethanolic extract). Quantification of the detected metabolites was assessed based on the relative response compared to internal standard adonitol and expressed as relative abundance.

**Organic acids**		
**Lactic acid**	**1.02**	relative abundance compared to the internal standard (adonitol)
**Malic acid**	0.44	
**Erythronic acid**	0.23	
***D*-arabinonic acid**	0.28	
**Xylonic acid**	1.93	
***L*-(+)-tartatic acid**	2.67	
**Ribonic acid**	6.43	
**Palmitic acid**	0.65	
**Sugars**		
***D*-(+)-xylose**	0.32	relative abundance compared to the internal standard (adonitol)
***D*-(-)-fructose**	5.80	
***D*-(-)-Tagatose**	4.46	
***d*-Glucose**	22.47	
***d*-Galactose**	3.72	
**Sucrose**	109.77	
***D*-Mannitol**	0.26	
**Myoinositol**	2.95	
**Glucerol**	3.74	
**Other organic molecules**		
**Gluconic acid**	0.20	relative abundance compared to the internal standard (adonitol)
***D*-(-)-Tagatofuranose**	8.90	
***D*-glucopyranoside**	8.50	

***D*-Galactopyranoside**	0.60

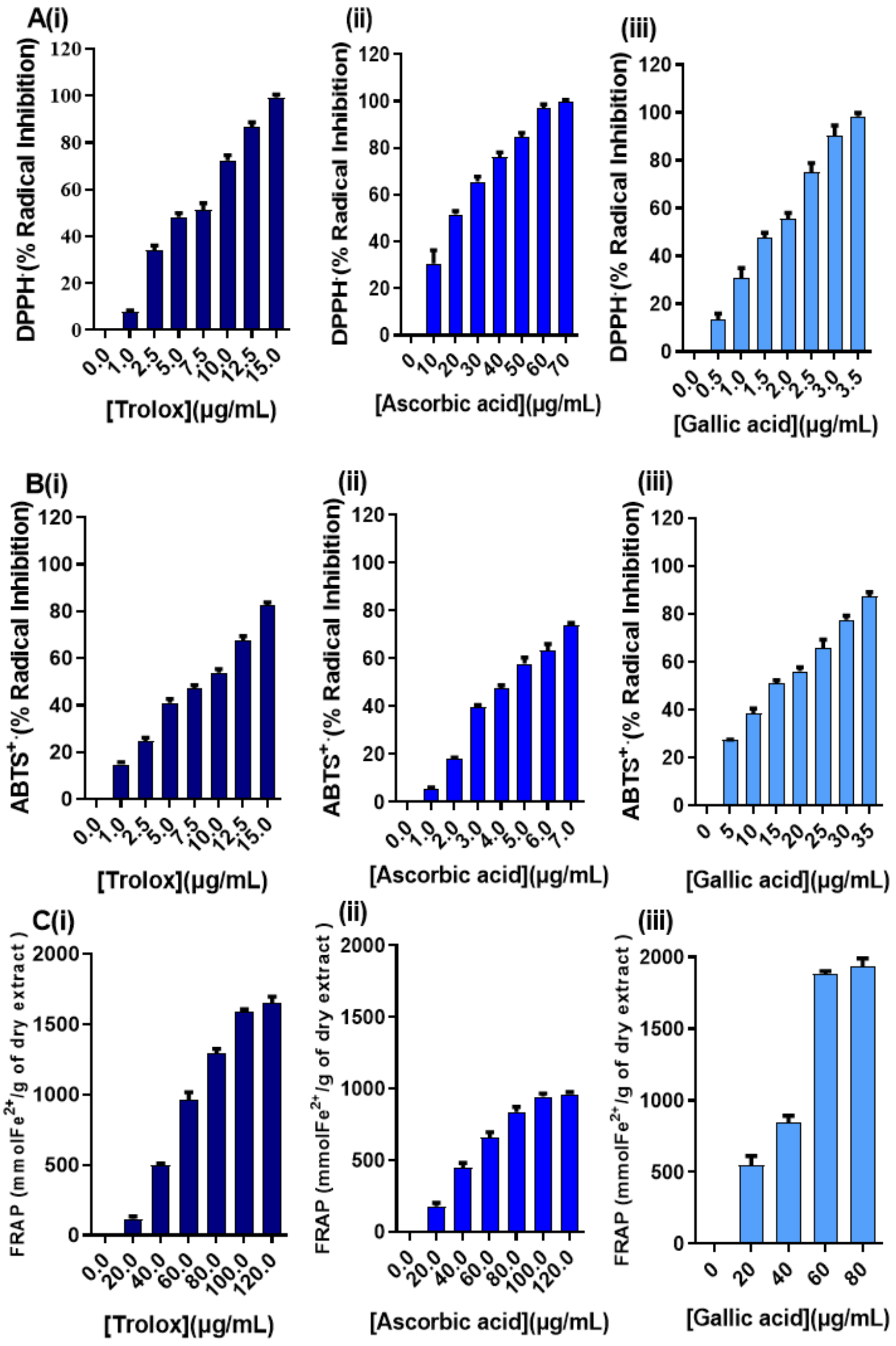

Figure S5. Antioxidant capacity of the positive controls; Trolox, Ascorbic acid and gallic acid as determined by (**A**) DPPH•, (**B**) ABTS•+ and (**C**) FRAP assay. Data are expressed as means ± SEM and are representative of three independent experiments.
